# u-PAR expression in cancer associated fibroblast: new acquisitions in multiple myeloma progression

**DOI:** 10.1186/s12885-017-3183-y

**Published:** 2017-03-24

**Authors:** S Ciavarella, A Laurenzana, S De Summa, B Pilato, A Chillà, R Lacalamita, C Minoia, F Margheri, A Iacobazzi, A Rana, F Merchionne, G Fibbi, M Del Rosso, A Guarini, S Tommasi, S Serratì

**Affiliations:** 1 National Cancer Research Centre IRCCS “Giovanni Paolo II”, 70124 Bari, Italy; 2 Department of Experimental and Clinical Biomedical Sciences, Section of Experimental Pathology and Oncology, University of Florence, Florence, Italy; 3 Molecular Genetics Laboratory, National Cancer Research Centre, IRCCS “Giovanni Paolo II”, 70124 Bari, Italy; 4 Nanotecnology Laboratory, National Cancer Research Centre, IRCCS “Giovanni Paolo II”, Bari, Italy

**Keywords:** u-PAR, Cancer-associated fibroblasts (CAF), Fibroblast activation, Multiple myeloma microenvironment

## Abstract

**Background:**

Multiple Myeloma (MM) is a B-cell malignancy in which clonal plasma cells progressively expand within the bone marrow (BM) as effect of complex interactions with extracellular matrix and a number of microenvironmental cells. Among these, cancer-associated fibroblasts (CAF) mediate crucial reciprocal signals with MM cells and are associated to aggressive disease and poor prognosis. A large body of evidence emphasizes the role of the urokinase plasminogen activator (u-PA) and its receptor u-PAR in potentiating the invasion capacity of tumor plasma cells, but little is known about their role in the biology of MM CAF. In this study, we investigated the u-PA/u-PAR axis in MM-associated fibroblasts and explore additional mechanisms of tumor/stroma interplay in MM progression.

**Methods:**

CAF were purified from total BM stromal fraction of 64 patients including monoclonal gammopathy of undetermined significance, asymptomatic and symptomatic MM, as well as MM in post-treatment remission. Flow cytometry, Real Time PCR and immunofluorescence were performed to investigate the u-PA/u-PAR system in relation to the level of activation of CAF at different stages of the disease. Moreover, proliferation and invasion assays coupled with silencing experiments were used to prove, at functional level, the function of u-PAR in CAF.

**Results:**

We found higher activation level, along with increased expression of pro-invasive molecules, including u-PA, u-PAR and metalloproteinases, in CAF from patients with symptomatic MM compared to the others stages of the disease. Consistently, CAF from active MM as well as U266 cell line under the influence of medium conditioned by active MM CAF, display higher proliferative rate and invasion potential, which were significantly restrained by u-PAR gene expression inhibition.

**Conclusions:**

Our data suggest that the stimulation of u-PA/u-PAR system contributes to the activated phenotype and function of CAF during MM progression, providing a biological rationale for future targeted therapies against MM.

## Background

Multiple myeloma (MM) is an incurable haematological malignancy in which plasma cells (PC) progressively expand as effect of sequential genetic changes and multiple interactions with bone marrow (BM) microenvironment [1]. The common clinical evolution from monoclonal gammopathy of undetermined significance (MGUS) toward asymptomatic and symptomatic active MM is underlain by a multistep process in which both plasma cells and their local microenvironment undergo progressive changes that, in turn, foster growth and survival of transformed cells [2]. Fibroblasts are a prominent cellular component of BM microenvironment and, under the influence of tumor and inflammatory factors, acquire a persistent state of activation, which is typical of the cancer-associated fibroblasts (CAF) [3]. CAF typically display enhanced expression of structural proteins as alpha-smooth muscle actin (α-SMA), vimentin and fibroblast specific protein 1 (FSP-1), and secrete high amounts of cytokines and growth factors directly involved in MM pathogenesis, such as interleukin 6 (IL-6), vascular endothelial growth factor (VEGF), transforming growth factor-beta (TGF-β), fibroblast growth factor-2 (FGF-2), and hepatocyte growth factor (HGF). They also gain improved capacity of extracellular matrix (ECM) degradation by over-producing metalloproteinases (MMPs) and other enzymes [4], which favours the expansion of PC clone within the BM [5].

Conversion of plasminogen into the broad-spectrum protease plasmin is known to contribute to ECM degradation in both solid and hematologic tumors such as MM [6–8]. This process, in turn, depends on the activation of the urokinase-type plasminogen activator (u-PA) system, which is composed by the proteinase u-PA, its receptor u-PAR and two major plasminogen activation inhibitors (PAI-1 and -2) [9]. The u-PA system mediates cell invasion [10, 11], the MMP-dependent BM degradation [12, 13], and stimulates the production of MM-supporting factors, such as HGF, by BM stromal cells [14]. Increasing evidence suggests a critical relation between u-PA system activation and MM aggressiveness and high levels of soluble u-PAR were described as an independent negative prognostic factor in MM [15, 16]. However, despite most research focused on the role of this system in cancer cells, very little is known about its function in CAF as pivotal controllers of MM progression.

We hypothesized that the activation state of CAF in MM may also involve a peculiar modulation of the u-PAR-controlled proteolytic activity of these cells. Thus, we explored the grade of functional activation of CAF from different clinical phases of the disease, in relation to both expression and function of major uPA system components. Intriguingly, we observed that CAF from patients with active disease show a significant up-regulation of the fibrinolytic system, prompt U-266 cell line invasivity and exert themselves higher proliferative and invasion capacity, which are dramatically restrained by a selective *u-PAR* silencing.

## Methods

### Patients and cell cultures

BM aspirates were obtained for diagnostic purpose from 64 subjects including 18 MGUS, 14 asymptomatic MM (aMM), 20 symptomatic active MM (sMM), and 12 MM in remission phase (rMM). sMM patients had a median age of 72 years, they were newly diagnosed and treatment-naïve. The median age was 75 years for both MGUS and aMM groups, whereas rMM patients had a median age of 79 years and were all in complete remission. All patients provided their written informed consent in accordance with the Declaration of Helsinki and this study was approved by the ethics committee of the National Cancer Research Institute IRCCS “Giovanni Paolo II” of Bari, Italy.

BM mononuclear cells (BMMCs) were separated by Ficoll-Hypaque gradient from 10 ml of heparinized BM aspirate for each sample. BM stromal cell (BMSCs) were isolated by plastic adherence of BMMCs on polystyrene flasks in DMEM medium (Euroclone, Milan, Italy) supplemented with 10% fetal bovine serum (FBS, Sigma, St. Louis, MO, USA) for about 2 weeks. Fibroblasts were then purified from BMSCs by immunoselection using D7-FIB-conjugated (anti-fibroblasts) microbeads (MiltenyiBiotec, Italy). Briefly, up to 10^7^ cells were centrifuged (300Xg) for 10′ and then resuspended in a Phosphate-Buffered Saline solution (Euroclone, Milan, Italy) added with 0.5% Bovine Serum Albumin (Sigma, St. Louis, MO, USA), and 2 mM sEDTA (Sigma, St. Louis, MO, USA). The cells were incubated at room temperature (RT) in the presence of Anti-Fibroblast MicroBeads for 30 min, then washed, centrifuged (300Xg) for 10 min and resuspended in 500 μL of buffer to undergo immunomagnetic positive separation in autoMACS Pro Separator (Miltenyi Biotec, Italy), according to manufacturer protocols. The proportion of CAF obtained after separation ranged between 4 × 10^5^ and 1 × 10^6^ cells/sample. Once isolated, CAF were expanded (until passage 4) in 10%FBS-added DMEM and stored for subsequent experiments.

U-266 human myeloma cell line was purchased from ATCC (LGC Standards, UK) and cultured in RPMI-1640 medium (Euroclone, Milan, Italy) supplemented with 10% FCS.

### Cell phenotype characterization

CAF were analyzed in whole heparinized BM aspirate from each patient sample. The cells were treated with lysing solution to eliminate erythrocytes and incubated with a mouse anti-human αSMA-FITC monoclonal antibody (Abcam, Cambridge, UK) and a rabbit anti-human FSP-1 antibody (Sigma-Aldrich, St Louis, MN, USA), an anti-CD45 (Becton Dickinson (San Josè, CA, USA). Corresponding cell surface antigens were detected by flow cytometryusing a FACSCanto cytometer (Becton-Dickinson) with the FACSDiva software (Becton-Dickinson) and the negative controls included isotype-matched antibodies. CAF were enumerated by quantifying the proportion of α-SMA/FSP-1 co-expressing cells within the gated CD45-negative cells, as reported [3].

### u-PAR gene silencing

*u-PAR* expression was inhibited in CAF by the anti-messenger 18-mer oligodeoxynucleotidephosphodiester aODN42 (50-CGG CGG GTG ACC CAT GTC-30) and a degenerated 18-mer ODN (dODN) (ISIS Pharmaceuticals, Carlsbad Research Center, CA, USA) was used as negative control. Uptake and stability of ODN were enhanced by combining ODNs (10 mM/l) with the cationic liposome solution DOTAP (13 mM/l) (Boehringer Mannheim, Mannheim, Germany), as described [17].

CAF preparations from each sample were cultured at 10 X 10^4^/plate for 72 h (h) in absence or presence of either 10 mM DOTAP-combined aODN or dODN in DMEM containing 10% heat-inactivated FCS. Based on ODN half-life in culture, a second addition of 5 mMODNs was performed after 24 h and 48 h in CAF culture. Cell viability were monitored daily by Trypan blue staining and non-adherent cells enumerated at each DOTAP/ODN addition and neither viability impairment nor detached cells were observed. Real-time reverse transcriptase polymerase reaction (RT-PCR) was used to monitor changes in *u-PAR* expression, which appeared steady reduced after 3-day exposure to aODN. Thus, at day 4, cells were collected and used for subsequent analyses.

### Proliferation studies

CAF (1 × 10^5^/sample) were plated in T25 flasks and incubated with DMEM plus 10% fetal bovine serum (FBS, Sigma, St. Louis, MO, USA) for 72 h. Then, they were detached by EDTA at different time points (24, 48, and 72 h) and counted by a cell counter. Similar experiments were performed on CAFs in absence or presence of ODNs, to evaluate whether *u-PAR* expression affects their replication activity. Moreover, to investigate the effect of CAF on MM cell proliferation, U266 cells were cultured in RPMI medium (8 × 10^4^/well) for 96 h in presence or absence of CM from sMM CAF either treated or not with anti-uPAR aODN. At different time points (24, 48, 72 and 96 h), the cells were resuspended and counted as described.

### Western blotting

U266 cells were incubated for 96 h in CM from sMM CAF previously treated with DOTAP, aODN or dODN, and then lysed in a 10 mM Tris-HCl buffer solution. 50 μg of the protein extract was electrophoresed in SDS-10% polyacrylamide gel and then blotted to a polyvinylidene difluoride membrane (Hybond-C Extra; Amersham Biosciences) for 3 h at 35 V. The membrane was blocked with 5% skim milk in 20 mM Tris buffer for 1 h at RT and then incubated overnight with primary antibodies against proliferating cell nuclear antigen (PCNA, Cell Signaling, Beverly, MA, USA) (1:2000) and β-Actin (1:2000) (SIGMA, St. Louis, MO, USA). After incubation with horseradish peroxidase-conjugated donkey anti-mouse IgG (Amersham Bioscience, GE Healthcare Europe GmbH, Milan, Italy) at 1:5000 dilution for 1 h, the immune-complexes were detected by the enhanced chemiluminescence ECL™ detection system (Amersham Bioscience, GE Healthcare Europe GmbH, Milan, Italy).

### Immunofluorescence

CAF were cultured on coverslips in DMEM medium in absence or presence of ODNs for 72 h, then fixed in 4% paraformaldehyde (Sigma-Aldrich, Italy), permeabilized in 0.1% Triton X-100 (Sigma-Aldrich, Italy), washed twice with PBS, and treated with primary fluorescent mouse-anti-u-PAR (1:40 dilution, Santa Cruz, CA, USA), mouse-anti-α-SMA (1:800 dilution; Sigma-Aldrich, Italy), and mouse-anti-Vimentin (1:800 dilution; Sigma-Aldrich, Italy) antibodies. Anti-mouseCy3–conjugated IgG, (1:800 dilution; Sigma-Aldrich, Italy), and anti-mouse FITC-conjugated IgG (1:800; Sigma-Aldrich, Italy) were used as secondary antibodies. Nuclei were stained with DAPI. The cells were observed by a fluorescence microscope (Leyca DC-200, Leyca Microsystem Imaging Solutions Ltd., Cambridge, UK) fitted with a digital camera.

### Real-time reverse-transcriptase polymerase chain reaction (RT-PCR)

Gene expression profile was assessed from total RNA extracted from CAF of MGUS and MM patients affected either treated or not with anti-uPAR aODN, as previously detailed. Complementary DNA was prepared from 1 μg of total RNA using a GoScript reverse transcription system (Promega, Italy). The relative quantity of urokinase plasminogen activator (u-PA), u-PA receptor (u-PAR), urokinase plasminogen inhibitor (PAI-1), MMP2, α-SMA and Vimentin messenger RNA was measured using the Applied Biosystems 7500 Fast Real-Time PCR System and calculated with the comparative Ct method using 18S ribosomal RNA as the normalization gene. Amplification was performed with the default PCR setting: 40 cycles of 95 °C for 15 s and of 60 °C for 60″ using SYBR Green-based detection (GoTaqqPCR Master Mix; Promega, Italy). The sequences of specific primers were reported in Table 1.

**Table 1:** Primer sequence and qPCR setting

Gene	Forward primer	Reverse primer
u-PA	5′-AGTGTCAGCAGCCCCACT-3′	5′-CCCCCTGAGTCTCCCTGG-3’
u-PAR	5′-GCCCAATCCTGGAGCTTGA-3’	5′-TCCCCTTGCAGCTGTAACACT-3’
PAI-1	5′-CTCCTGGTTCTGCCCAAGTT-3’	5′-GAGAGGCTCTTGGTCTGAAAG-3’
MMP-2	5′-AGCACCGCGACAAGAAGTAT-3’	5′-ATTTGTTGCCCAGGAAAGTG-3’
18S rRNA	5′-CGGCTACCACATCCAAGGAA-3’	5′-GCTGGAATTACCGCGGCT-3’
α-SMA	5′-CTGTTCCAGCCATCCTTCAT-3’	5′-CCGTGATCTCCTTCTGCATT-3’
Vimentin	5′-TGTCCAAATCGATGTGGATGTTTC-3’	5′-TTGTACCATTCTTCTGCCTCCTG-3’

PCR setting: 40 cycles of 95 °C for 15 s and of 60 °C for 60 s using SYBR Green–based detection (GoTaqqPCR Master Mix; Promega)

### Invasion assay

Invasion capacity of CAFs was evaluated either in absence or presence of ODNs, as previously described [17]. Briefly, 8 μm–pore size polycarbonate filters (BD Biosciences, Italy) were coated with Matrigel (BD Biosciences, Italy) and placed in Boyden chambers. 8 × 10^3^ cells were plated onto the upper side of the chamber. After 18 h–incubation at 37 °C, the filters were fixed by methanol and the cells on the upper surface removed by a cotton swab. The cells migrated onto the lower side of the filter were stained by Diff-Quick (Mertz-Dade AG, Dade International, Milan, Italy) and counted by light microscopy (40X magnification). Similarly, the invasion ability of U266 cells was also evaluated. 1 × 10^4^ cells were plated onto the upper side of the Boyden chamber in absence or presence of CM from sMM CAF either treated or not with ODNs. After 6 h–incubation the number of cells moved across the filter was assessed. Experiments were performed in triplicate. Invasion was expressed as mean ± SD of the number of total cells counted/filter.

### Statistical analysis

The differences in the values of parameters among the groups were examined by ANOVA and Tukey’s Multiple Comparison Test as post hoc analysis through GraphPad Prism version 5.00 for Windows (GraphPad Software, San Diego California USA, www.graphpad.com). Results of qRT-PCR were expressed as median with range. *p*-value <0.05 was considered statistically significant.

## Results

### Frequency and activation state of CAF vary among MM progression

**Fig. 1:**
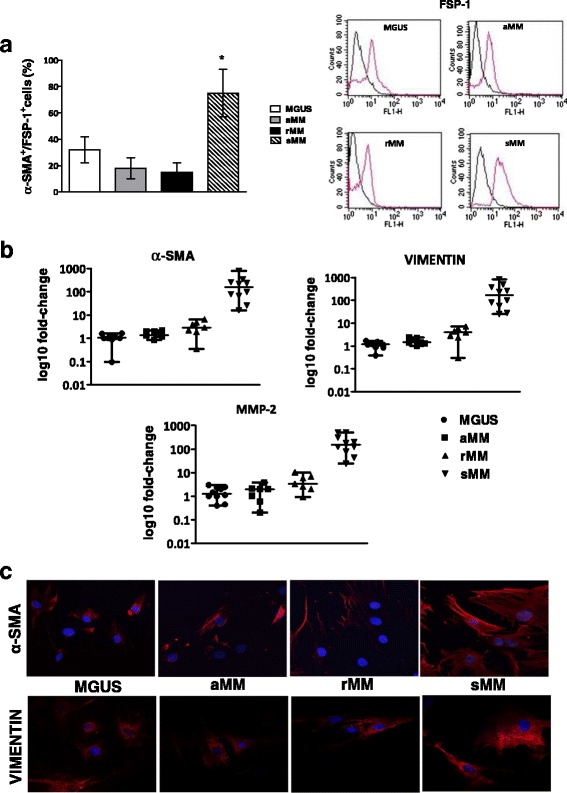
**a** Flow cytometry analysis of CAF frequency (αSMA/FSP-1 positive cells on gated CD45- population) in patients with MGUS, aMM, rMM and sMM. Data are reported as mean ± SD from three different experiments performed in triplicate (*p < 0.01). On the right, representative flow cytometry histograms of FSP-1 positive cells for each study group. **b** Quantitative PCR analysis of α-SMA, Vimentin and MMP-2 in CAF. Results were obtained in 3 different experiments performed in triplicate. **c** Representative immunofluorescence panels of α-SMA (red) and Vimentin (red) in CAF from MGUSand MM patients at different disease phase (600X magnification)

**Fig. 2:**
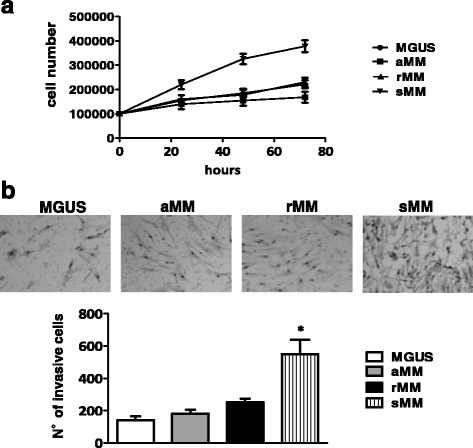
**a** Proliferation assays of CAF plated at 10 × 10^4^/flask and enumerated after 24, 48 and 72 h of culture. Each value is the mean number of cells ± SD from three different experiments performed in triplicate. **b** 6-h Matrigel invasion assay. Invasive CAF were counted in each filter by light microscopy. Each value is the mean number of cells ± SD of three different experiments performed in triplicate (*p < 0.05)

As depicted in Fig. 1a, flow cytometry analysis demonstrated that CD45^−^ cells co-expressing FSP-1 and α-SMA, as major phenotypic markers of CAF, are significantly increased in the BM of patients with sMM (75 ± 18%) compared to MGUS (32 ± 10%), aMM (18 ± 8%), rMM (15 ± 7%). Of note, transcription of α-SMA, vimentin and MMP-2 genes was up-regulated in CAF from patients with more active disease, since their fold-change was significantly higher in sMM patients with respect to MGUS, aMM and rMM (*p* < 0.0001 in all instances) (Fig. 1b). These data support the idea that fibroblasts at different phase of the disease are characterized by diverse grades of activation. By immunofluorescence, we also observed that CAF from patients with sMM express αSMA, a major marker of functional activation, at much more intensity than MGUS, aMM and rMM, as shown in the representative panels of Fig. 1c. Consistently, CAF from patients with active symptomatic disease showed a remarkable proliferative capacity in vitro. Their absolute number rapidly increased during a 3-day culture period and, after 72 h, was approximately two-fold higher than CAF from MGUS, aMM and rMM, exposed to similar culture conditions (Fig. 2a). When incubated in a Boyden chamber system, moreover, CAF showed a peculiar invasion potential, which was significantly pronounced in CAF from sMM as compared to others. Figure 2b exemplifies this difference, showing the higher number of sMM CAF migrated toward Matrigel-coated filters.

### Expression of the fibrinolytic system components by MM-associated fibroblasts

**Fig. 3:**
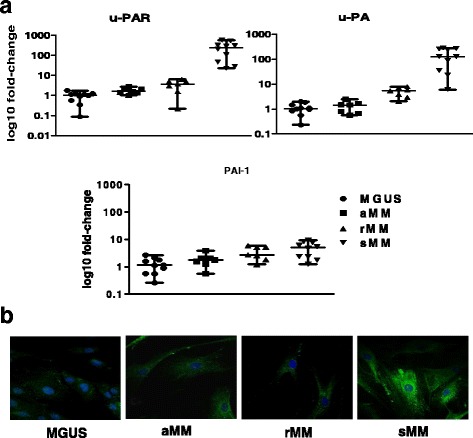
**a** Quantitative PCR analysis of uPA, uPAR, and PAI-1 in CAF from MGUS and MM patients. Data shown were obtained in 3 different experiments performed in triplicate. **b** Representative immunofluorescence panels of u-PAR (green) in CAF (600X magnification)

We investigated by RT-PCR the transcriptomic profile of the major members of the fibrinolytic system and interestingly found that CAF from patients at diverse phase of MM progression also show a differential and peculiar expression of *u-PAR, u-PA,* and *PAI-1*. As can be seen in Fig. 3a, CAF from active sMM hold the highest transcription level of *u-PAR* and *u-PA,* showing a significant up-regulation of these genes as compared to MGUS, aMM, and rMM, in which their level was globally low. Similarly, PAI-1 expression was weak and no significant difference was appreciable between groups. These data were in part supported by immunofluorescence experiments exploring the expression of u-PAR at protein level. Representative UV microscopy images in Fig. 3b depict distinctive patterns of u-PAR exposition, appearing as green fluorescent spots on membranes of CAF from different moments of MM progression. A dramatic increase of the u-PAR signal was evident in CAF from sMM with respect to MGUS and aMM, whose florescence was comparable to rMM.

### u-PAR silencing affects CAF phenotype and function in MM

**Fig. 4:**
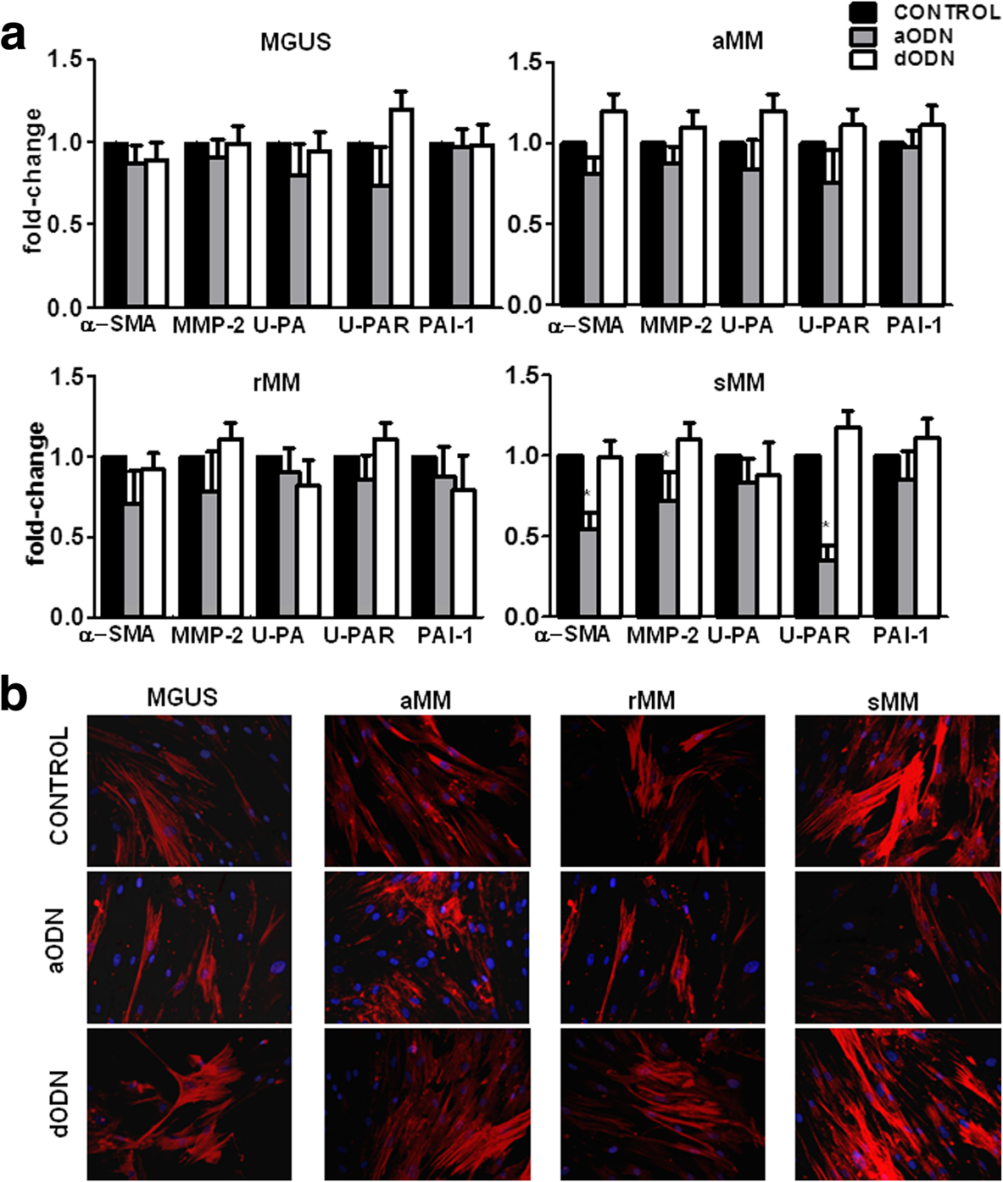
**a** Quantitative PCR analysis of uPA, uPAR, PAI-1, α-SMA and MMP-2 in u-PAR-silenced CAF from MGUS and MM patients. Data were obtained from 3 different experiments performed in triplicate (*p* < 0.0001). **b** Representative panels of immunofluorescence staining of α-SMA (red) in u-PAR-silenced CAF (600X magnification)

To explore whether u-PAR contributes to the acquisition of an activated phenotype by fibrobalsts in MM, we studied how selective u-PAR silencing influences the expression of α-SMA, MMP-2, u-PA and PAI-1 in CAF from MGUS, aMM, sMM and rMM. As shown in Fig. 4a, the exposure to the antisense aODN resulted in a relevant decrease of u-PAR expression in all study groups, and particularly in cells from sMM. All u-PAR-silenced cells, moreover, showed a consensual down-regulation of α-SMA, and MMP-2 genes and this effect was significant in CAF from sMM. U-PA underwent similar down-modulation, although its decrease in u-PAR-silenced cells reached no significant values in all CAF preparations. Immunoflurescence experiments emphasized, almost in part, these data, since after ODN treatment, α-SMA signal appeared considerably attenuated in CAF from sMM, while those from MGUS, aMM and rMM showed comparable levels of the protein either before or after inhibition of u-PAR gene expression (Fig. 4b).

**Fig. 5:**
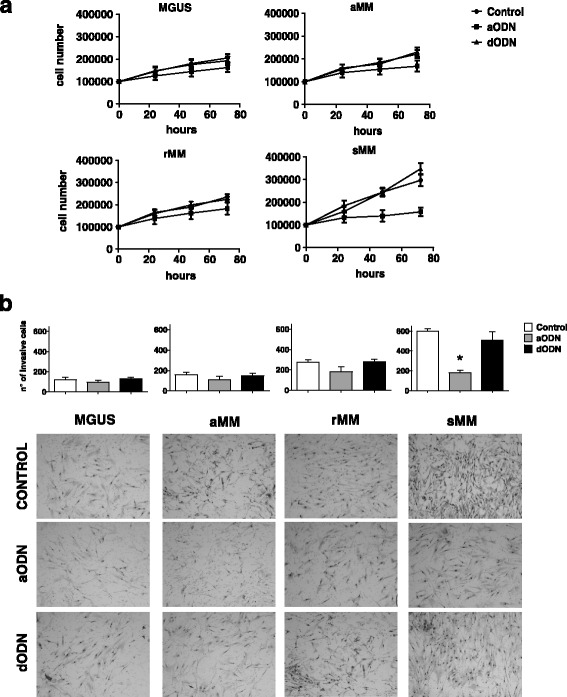
**a** Proliferation and (**b**) invasion assays of u-PAR-silenced CAF from patients with MGUS, aMM, rMM and sMM (*p* < 0.01). Representative optical microscopy images of Matrigel-invasive CAF are shown in the lower side of the figure

Furthermore, proliferation and invasion assays performed before and after u-PAR silencing were aimed at exploring whether the receptor is implicated in crucial functions of activated CAF. Figure 5a illustrates that u-PAR silencing dramatically impaired the growth kinetics of CAF from sMM, since after exposure to aODN they underwent a two-fold decrease of their proliferation over 72 h as compared to controls. No significant modification of the proliferative behavior was noted in CAF from MGUS, aMM and rMM. Moreover, while these latter showed no changes in their Matrigel invasion capability after u-PAR inhibition, CAF from sMM resulted significantly restrained in their invasive attitude, since the number of u-PAR-silenced cells migrated toward filters was up to three fold inferior to the controls (Fig. 5b). These data provide support to the hypothesis that u-PAR/u-PA system is critically involved in the invasive function of CAF during MM progression.

### uPAR modulation on CAF affects MM cell proliferation and invasion potential

**Fig. 6:**
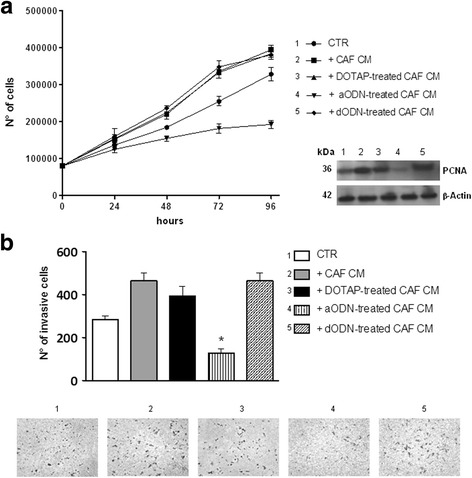
**a** Proliferation assay of U266 myeloma cells enumerated at 24, 48, 72, and 96 h of culture in normal medium (CTR) or in conditioned medium (CM) from sMM untreated, DOTAP-, aODN-, and dODN-treated CAF. Western blot image shows the expression of the proliferation cell nuclear antigen (PCNA) in U266 cells at 96 h of incubation in the above conditions. **b** Graph depicting the quantification of U266 cells migrated under the stimulus of normal medium (CTR) or medium conditioned by sMM untreated, DOTAP-, aODN-, and dODN-treated CAF (*p < 0.05). Representative optical microscopy images for each culture condition of the invasion assay

In order to investigate whether or not CAF influence MM cell properties in relation to u-PAR expression, we carried out both proliferation and invasion assays by stimulating U-266 cells with medium conditioned by primary sMM CAF either treated or not with anti-u-PAR aODN. As shown in Fig. 6a, U-266 cells under the stimulus of CM from sMM CAF showed higher proliferation capacity than control even at 48 h and, more significantly, at 72 h of culture. On the contrary, their proliferation was dramatically reduced in CM from u-PAR-silenced CAF, since at 96 h of culture their absolute number was about 3-fold lower than those incubated in active CAF CM. Results from Western blot analysis supported these data since the protein expression of the proliferating cell nuclear antigen (PCNA) in U266 cells paralleled their proliferation behavior after 96 h of incubation.

Moreover, as depicted in Fig. 6b, MM cells displayed a considerable inherited attitude to invade and migrate toward porous Matrigel-coated filters in vitro and this property was significantly improved by incubating the cells in CM from sMM CAF. Notably, when the invasion experiments were performed using the CM from uPAR-silenced CAF, the number of U266 cells migrated on the bottom side of the Boyden chamber was about 2-fold and more than 3-fold lower than those cultured in normal medium and in CM from sMM CAF, respectively.

## Discussion

The present study focuses on the role of CAF in the biology of MM progression and emphasizes the contribution of the u-PAR-driven fibrinolytic system to the process of MM cell expansion within the BM.

It is well accepted that chronic interactions between malignant PCs and surrounding cells sustain a progressive expansion of the tumor clone within the BM, driving the clinical transition from MGUS to overt MM. Bone degradation/invasion represents a crucial step of this process and is mainly ascribed to the enhanced capacity of tumor cells to produce digestive enzymes as MMPs and other pro-invasive molecules, disrupt ECM proteins, and respond to a number of chemotactic stimuli by both inflammatory and stromal cells within the BM [5]. On the other hand, adhesion to ECM proteins such as fibronectin and laminin, and stromal elements as osteoblasts, endothelial cells and particularly fibroblasts, prompt myeloma cell survival and drug resistance [18], further promoting the disease progression. In this scenario, a fraction of BM fibroblasts, known as CAF keeping a chronic state of functional activation is thought to actively support tumor progression [3].

Our data are in line with these observations, since CD45^−^/α-SMA^+^/FSP-1^+^ cells showing a typical CAF phenotype more largely infiltrate BM of patients with active MM than MGUS, aMM and rMM, suggesting that CAF expansion parallels MM growth. Consistently, gene expression and immunofluorescence analyses indicated that CAF in symptomatic MM patients express significant higher levels of structural proteins as α-SMA and vimentin, recognized as fibroblast activation markers, and the metalloproteinase MMP-2. The condition of prominent activation also impacts the invasive and proliferative ability of fibroblasts, which are much more pronounced in the sMM compared to the other stages of the disease. These observations emphasizing the hypothesis that CAF in MM, as in other solid neoplasm, derive from enhanced commitment of normal fibroblasts to more activated myofibroblasts with improved ECM-degrading potential [19]. At this regard, recent studies reported that MM cell interaction with tumor stroma results in a critical increase of u-PA and other ECM protein levels, stressing the role of the fibrinolytic system in the MM-associated bone destruction and progression [20, 21]. Moreover, binding of u-PA to its receptor strongly accelerates the enzymatic activity of u-PA itself and also described to promote angiogenesis [22, 23]. Our findings intriguingly indicated that, as compared to MGUS, aMM and MM in remission phase, CAF from sMM significantly overexpress both u-PA and u-PAR genes as key components of the fibrinolytic system, and display higher level of u-PAR on cell surface. This may be ascribed to the increased BM concentrations reached in active MM by a number of inflammatory factors produced by stromal, immune and tumor cells themselves as effect of multiple reciprocal paracrine and cell-to-cell interactions. Some of these, such as TGF-β and SDF-1, are indeed reported to mediate the up-regulation of the u-PA/u-PAR axis [6, 24] as well as the switch of CAF to a strongly activated phenotype [3].

In parallel with a strongly activated phenotype, BM-infiltrating CAF in active MM rather than MGUS, asymptomatic and treated patients, show a peculiar pro-invasive asset, which also support bone matrix degradation and plasma cell invasion. The idea that activation of the u-PA/u-PAR system is connected to the global triggering of CAF function in active MM is further suggested by results from our u-PAR silencing experiments. In fact, inhibition of u-PAR mRNA by selective anti-sense oligonucleotides produced a consensual down-regulation of u-PA and MMP-2 expression as well as of α-SMA*,* at both mRNA and protein level, and this effect was particularly evident in active MM as compared to other clinical phases of the disease. It is thus conceivable that, as the disease progresses, structural and functional changes in BM fibroblasts also involve, and are interconnected with, modulation of the u-PA/u-PAR axis components, although further research is needed to identify specific mechanisms of reciprocal intracellular signal control. At functional level, we observed that more intense proliferative and invasion attitude characterizes CAF expressing higher levels of u-PAR as those from active symptomatic MM and, notably, these properties were dramatically restrained by u-PAR silencing. Finally, in our in vitro experiments CAF showed to affect MM cell functions by a paracrine mechanism since malignant cells significantly increased their replication and invasion ability under the stimulus of CM from sMM CAF, while this effect was dramatically lowered by incubation in CM from u-PAR-silenced CAF. Although further investigation is needed to better characterize the role of u-PA/uPAR system in MM, our findings provide insights about it as a critical part of the complex network of paracrine and physical signals implicated in the biology of MM progression.

## Conclusions

To the best of our knowledge, this is the first report in which the molecular role of u-PAR/u-PA system emerges as directly implicated in the biology of CAF and interconnected with the pathophysiology of MM progression. Based on these and indirect evidences from others, u-PAR may be recognized as an additional marker of CAF activation in MM and a key molecular player of their capability to promote bone matrix destruction and ECM degradation, thus favoring migration and invasive properties of malignant plasma cells. In this view, the use of novel anti-u-PAR compounds, including antagonist peptides and monoclonal antibodies as well as emerging gene-silencing approaches [25], acquires a robust rationale in MM and might be effective in targeting, at the same time, both malignant cells and tumor microenvironment.

## Abbreviations

aMM: Asymptomatic multiple myeloma
BM: Bone marrow
BMMC: Bone marrow mononuclear cell
BMSC: Bone marrow stromal cell
CAF: Cancer associated fibroblast
ECM: Extracellular matrix
FBS: Fetal bovine serum
FSP1: Fibroblast-specific protein 1
IGF1: Insulin-like growth factor 1
IL-6: Inteleukin 6
MGUS: Monoclonal gammopathy of undetermined significance
MM: Multiple Myeloma
MMP2: Matrix metalloproteinase 2
PAI-1: Plasminogen activator inhibitor-1
PC: Plasma cell
rMM: Remission multiple myeloma
SDF1: S: Stromal cell-derived factor 1
sMM: Symptomatic multiple myeloma
TGFbeta: Transforming growth factor beta
TME: Tumor microenvironment
u-PA: Urokinase plasminogen activator
u-PAR: Urokinase receptor
α-SMA: alpha smooth muscle actin
